# Wetting and Interaction of Titanium Melt with Calcium Titanate

**DOI:** 10.3390/ma19010072

**Published:** 2025-12-24

**Authors:** Axaule Mamaeva, Alexander Panichkin, Bagdaulet Kenzhaliyev, Alma Uskenbayeva, Marzhan Chukmanova, Balzhan Kshibekova, Zhassulan Alibekov

**Affiliations:** Institute of Metallurgy and Ore Beneficiation, Satbayev University, Almaty 050010, Kazakhstan

**Keywords:** CaTiO_3_, titanium melt, wetting, reactive interaction, diffusion

## Abstract

This study presents the results of a study of the reaction interaction and contact angle during contact between a titanium melt and a calcium metatitanate substrate. It is shown that at temperatures slightly above the melting point, the titanium melt poorly wets the CaTiO_3_ substrate surface. The contact angle and the onset temperature of active reaction vary depending on the fractional composition of the CaTiO_3_ powders from which the substrates are made and their porosity. Under isothermal holding conditions below the onset temperature of active reaction, the contact angle changes insignificantly. At the onset temperature of the reaction interaction, after a short stabilization period of the contact angle, the reaction leads to rapid penetration of the molten droplet into the depth of the CaTiO_3_ substrate and, in some cases, to the expulsion of a Ca-Ti-O liquid phase from the reaction zone. The interaction of titanium melt with calcium titanate is accompanied by a series of physicochemical reactions associated with the reaction interaction, which intensifies with increasing temperature and causes the restoration of calcium to a metallic state and the dissolution of oxygen in the titanium melt, as well as the formation of a liquid Ca-Ti-O layer in the transition zone.

## 1. Introduction

Titanium and titanium alloys are widely used in various fields of technology due to their unique properties, but their production volumes are small due to high production costs. This is due to the relatively high cost of raw materials, the refractoriness of titanium alloys and the significant chemical activity of titanium melts during their production. Therefore, the production of titanium alloys requires special conditions for the preparation of charge, the use of energy-intensive furnaces that ensure the melting under vacuum conditions and in the absence of contact with refractory materials. It is achieved by the use of vacuum skull melting furnaces with electric arc or electron beam energy sources [1]. At the same time, another problem is the production of titanium products by casting the alloy. It is due to the high activity of melts also during pouring resulting in the formation of an alpha case on the surface of the casting and the formation of pores and other defects caused by the interaction of the melt with the walls of the casting mold from the moment the melt is poured until it solidifies [2]. As a consequence, there is a need to search for inert materials that do not actively interact with the titanium alloy during melting and casting. Therefore, further investigations are currently required to examine the interaction processes that develop upon contact between liquid titanium and refractory or molding materials of various compositions.

For many decades, studies have been conducted to find materials that are inert or relatively inert to titanium melts [1,2,3,4,5,6,7,8,9,10,11,12,13,14,15,16,17,18,19,20]. The interaction of titanium melts with a variety of simple and complex oxides, nitrides, carbides and graphite is studied within the framework of these studies. However, all these materials still cause an interfacial reaction, and the reaction between graphite and titanium alloy results in carburization and the formation of a carbonized layer that includes a carbon-rich brittle phase [3,4,5,6].

According to [2], various materials have been evaluated for use in the melting of titanium alloys, showing that relatively stable materials are oxides—Y_2_O_3_, CaZrO_3_, BaZrO_3_, etc. This observation supports the recommendation of these compounds as lining materials for induction furnaces and as materials for the fabrication of crucibles used in melting titanium alloys. However, it is well established that ZrO_2_ develops a reaction layer with the titanium-alloy melt at the solid–liquid interface. At the same time, Y_2_O_3_ ceramics, despite the best resistance to titanium melts, has a high cost and can be used only in low-tonnage production. Refractories and molding materials made of CaZrO_3_, BaZrO_3_ [7,8,9,10,11] are economically more attractive, although their cost is also high.

However, as shown by [12], CaZrO_3_ in contact with titanium melt still interacts and causes contamination of titanium melt with zirconium and oxygen.

It is shown in [7] that titanium melt interacts well with the BaZrO_3_ surface, dissolving zirconium with oxygen, and also reduces barium to a metallic state. It calls the prospects of using these materials in lining and molding mixtures of the titanium industry into question. At the same time, the interaction of liquid titanium with BaZrO_3_ and SrZrO_3_ as well as SrTiO_3_ titanate was considered in the same work [7]. It is shown that titanium dissolves zirconium and oxygen and reduces barium and strontium to a metallic state in contact with these ceramic materials. Barium and strontium evaporate due to the high vapor pressure at the experimental temperature, and cause the melt to splash or form a vapor layer that reduces the interaction rate of the melt with the ceramic.

The results presented in [21] are of interest. In this work, the possibility of using a crucible made of (Ca,Sr,Ba)ZrO_3_ for vacuum induction melting of NiTi alloy is considered. The presented crucible material sintering technology at 1500 °C results in a homogeneous distribution of elements. It was found after melting the titanium alloy in this crucible that the total oxygen and nitrogen content remaining in the TiNi alloy after (Ca,Sr,Ba)ZrO_3_ crucible melting was 0.0173% wt%, that is in line with the ASTM (American Society for Testing and Materials) standard for biomedical TiNi alloys. It is presented that the (Ca,Sr,Ba)ZrO_3_ crucible stability to molten NiTi is related to the slow diffusion effect of high-entropy ceramics. And the authors propose this material as a potential crucible material for melting titanium alloys in a vacuum induction furnace.

Considering the procedure for the selection and further use of potential materials for melting a particular titanium alloy, it should be noted that it is important to take several important aspects into account: the interaction of the material with the melt and the thermodynamics of the reactions involved, the melting and softening points of the refractory material, and wettability and heat resistance [2]. As a result, there is a need to study the processes of reaction diffusion and wetting that develop when titanium melts come into contact with the most inert materials, such as titanates and zirconates of alkaline, alkaline earth and rare-earth metals.

The works devoted to the general theory of wetting are extensive [13,14,15,16,17,18,19,20]. At the same time, metal wetting of ceramics is determined by two types of interactions occurring at the interface, leading to non-reactive wetting and reactive wetting [9,14]. Non-reactive wetting occurs in liquid/solid systems where mass transfer across the interface is very limited and has little effect on the interfacial energy. Wetting, involving the chemical alteration and/or diffusion of chemicals across the interface, is reactive wetting. It often occurs in metal/ceramic systems at high temperatures. However, only a small number of studies of interfacial phenomena and wettability of ceramic materials with titanium melts are reported in the literature.

For example, in [14], the wetting parameters and interaction of the surface of a yttria-stabilized zirconium dioxide plate with a pure titanium melt were examined. The experiments were conducted using the sessile drop method in an argon atmosphere at 1973 K. It was shown that interphase reactions occur at the melt/ceramic interface, and the contact angle increases with increasing substrate porosity. This is explained by the fact that porosity alters surface roughness. In the experiments, the contact angles were found to be stable and significantly exceed 90°. However, the authors noted the formation of a film of refractory TiC on the droplet surface during exposure. This indicates carbon evaporation from the surface of the graphite heater, which may indicate an erroneous measurement of the contact angle.

There are earlier works that studied the influence of titanium content in melts on the wetting of oxides in systems such as Ni–Ti/Al_2_O_3_ [22] and NiFeCr–Ti/Al_2_O_3_ [23]. These works examine the processes of wetting and spreading of liquids, where the microstructure and properties of the transition layer of contact between ceramics and metal are formed, which determines the properties of the system. It shows that there is a significant interaction between dissolved substances—O and Ti, causing adsorption of O–Ti clusters on the liquid side of the contact and the formation of metallic oxides, such as TiO, on the solid side of the contact interface [14]. And it apparently leads to a decrease in the contact angle.

Various studies [2,3,4,5,6,7,8,9,10,11,12] examine the interaction processes between liquid titanium and the zirconates and titanates of several alkaline-earth metals; however, the wetting process has not been addressed [24,25,26].

Ref. [12] describes the interaction of ceramic materials based on CaO and CaZrO_3_ with titanium melt. It is shown that when titanium melt interacts with CaZrO_3_, a highly porous CaTiO_3_ layer is formed in the reaction zone, while zirconium dissolves in the melt. No evidence of significant interaction between this layer and the titanium melt was detected. Based on this, the authors suggest that CaTiO_3_ could be used as a promising mold material for producing titanium alloy castings.

It can be noted that calcium metatitanate CaTiO_3_ is similar in properties to CaZrO_3_. It melts congruently at 1930 °C and exists in a strictly stoichiometric ratio. At high temperatures, it has a low vapor pressure [27]. It is known that when heated, CaTiO_3_ undergoes phase transitions from an orthorhombic structure at room temperature (Pbnm) to a tetragonal (I4/mcm) polymorphism, followed by a transformation into a cubic aristotype. The temperatures of these transformations, according to [28,29,30], differ and are 1373–1423 K or 1498 ± 25 K during the transition from an orthorhombic to a tetragonal lattice and 1523 ± 10 K or 1634 ± 13 K during the transition from a tetragonal to a cubic lattice. However, such phase transitions do not cause a significant change in the crystal lattice volume, as indicated by the results of the same studies. For example, during the I4/mcm → Pm $\bar{3}$ m transition, a jump in ΔV/V ≈ 0.5–0.6% is observed [30]. Over the entire range of 296–1720 K, the temperature dependence of the crystal lattice parameters is close to linear.

The use of CaTiO_3_ as a component of refractory and molding material for melting and casting titanium alloys is also considered in [31,32,33]. However, in [31], heating and holding of the titanium sample and ceramics was carried out at 1700 °C for 10 min in a graphite crucible, which caused the dissolution of carbon in the titanium and could affect the interaction at the interface between the melt and the ceramic composite. In [33], tests were conducted at 1600 °C, which is below the melting point of titanium.

In [34], based on the results of a study of interaction with titanium in the solid state at 1600 °C, a Y_2_O_3_/Ca_4_Ti_3_O_10_ composite was proposed as a material for creating barrier layers on the surface of casting molds to reduce their interaction with the titanium melt. The prospect of practical use of calcium metatitanate in the foundry production of titanium and its alloys is justified by the similarity of physical properties with Y_2_O_3_ and CaZrO_3_ (Table 1), as well as the significantly lower cost of this material in comparison with Y_2_O_3_ and CaZrO_3_ (17 and 3 times, respectively).

Since previous studies on the interaction of titanium melt with CaTiO_3_ ceramics were conducted under conditions that do not replicate the melting and casting conditions of titanium alloys, further studies on the interaction of CaTiO_3_ ceramics with titanium melt are necessary to evaluate the potential use of this compound in foundry applications. Under vacuum conditions and with the exclusion of carbon, it is necessary to study the reactions occurring at the solid–liquid interface. Furthermore, information on the effect of CaTiO_3_ ceramic porosity on the wetting properties of its surface by titanium melt is of practical interest.

In this regard, the aim of this work is to consider the processes developing during contact of titanium melt with calcium titanate in a vacuum by determining the wetting contact angle and studying the structure and distribution of elements in the transition zone.

## 2. Materials and Methods

It is known that the development of redox reactions and mutual diffusion is possible when titanates come into contact with titanium melt. As a result, new compounds in the form of single-phase, two-phase and three-phase layers can be formed at the interface of contact between the solid and liquid phases [1,2]. The limiting number of phases in the layers forming the diffusion zone under conditions of isothermal interaction, as it is known, is determined by the state diagram of the system. Intermediate phases that can form in the diffusion zone can also be judged based on the analysis of state diagrams of the corresponding systems [7].

**Figure 1:**
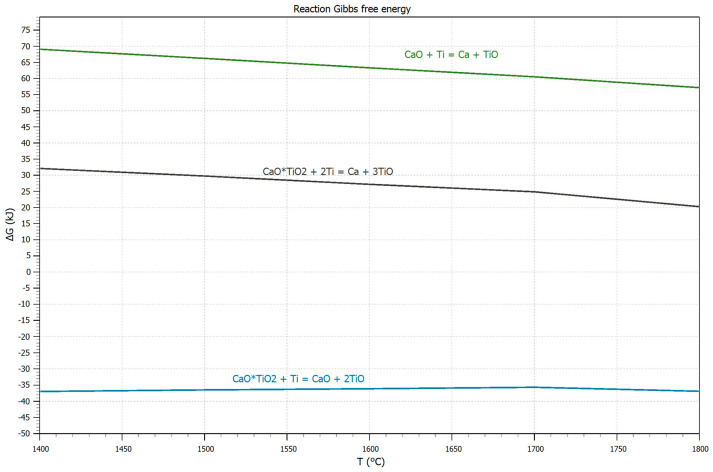
Temperature dependence of Gibbs energy for reactions (1)–(3).

The changes in Gibbs energy for reactions (1)–(3) were calculated to assess the possibility of interaction of titanium with calcium titanate. The “OUTOKUMPU HSC CHEMISTRY 8.0” was used for thermodynamic calculations. The results are presented in Figure 1.

CaTiO_3_ + 2Ti = Ca + 3TiO (1)

CaTiO_3_ + Ti = CaO + 2TiO (2)

CaO + Ti = Ca + TiO(3)

For reaction (1), the change in the Gibbs energy (∆G) in the temperature range from 1400 to 1800 °C is positive; hence, the reaction cannot proceed directly. It may indicate that the reaction of calcium titanate with titanium occurs in two stages.

According to reaction (2), calcium titanate should dissociate with separation of TiO_2_ and its dissolution in the melt as a result of interaction with titanium melt. At the same time, a calcium oxide layer should be formed at the contact boundary. It will form a protective barrier that prevents further interaction due to the positive Gibbs energy according to reaction (3). It allows us to expect that calcium titanate CaTiO_3_ can be an effective material to produce new refractory and molding materials for melting and casting of titanium alloys. It is characterized with high moisture resistance, resistance to interaction with carbon dioxide and low production cost. It makes it necessary to study the mechanism of interaction of calcium titanate with titanium melt experimentally.

Grade 2 was used (Table 2) and calcium titanate substrates were synthesized for an experimental study to determine the wetting angle and to consider the interaction of titanium melt with calcium titanate.

The calcium titanate was synthesized with the liquid-phase method under reaction (4).

CaCO_3_ + TiO_2_ = CaTiO_3_ + CO_2_ (4)

**Figure 2:**
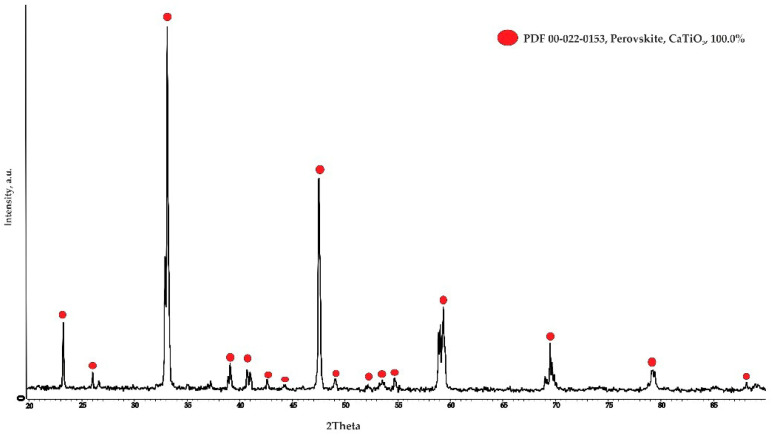
A diffractogram of the obtained CaTiO_3_ substrates.

In the first step, a suspension was obtained by mixing CaCO_3_ (99.9%) < 20 μm and titanium oxide TiO_2_ (99.5%) < 10 μm powders in water at a S:L ratio of 1/2 for a long time. The suspension was dried in a drying oven and then melted in a two-electrode arc furnace. The resulting sintered materials were crushed and re-melted to achieve a homogeneous composition. After double melting, the material was crushed again to obtain powders that were sized according to particle size. To determine the effect of substrate porosity on the reaction with the titanium alloy, powders with particle size distributions of ≤0.1 mm, 0.1 mm, 0.2 mm, 0.3 mm, and 0.5 mm were used. The resulting powders were mixed with an aqueous solution of distillery sulfide stillage added at the rate of 1% by weight. Tablets of ∅40 mm and a height of 5 mm were obtained from the mixture with a hydraulic press at a pressure of 2 MPa. These tablets were sintered at 1600 °C for 2 h in a normal atmosphere in a RHTV 120–600/C 40 “Nabertherm” tube furnace. The phase composition of the obtained ceramic tablets was studied using the D8 Advance X-ray diffractometer (BRUKER, Karlsruhe, Germany) with α-Cu radiation (λ~1.54 A). X-ray diffraction was performed with focusing using the Bragg–Brentano method. Diffractograms were recorded in the range of angles 2θ: 4–90° with a step of 0.05°, the shooting speed was 2 deg/min at a voltage of 40 kV and a current of 40 mA. The description and interpretation of the obtained results were carried out using the database of the Joint Committee for Powder Diffraction Standards—International Center for Diffraction Data (ICDD). The results obtained (Figure 2) confirm that the substrates are formed by CaTiO_3_ monophase.

The apparent density and porosity of tablets obtained with different powder fractions were measured. These data are presented in Table 3.

**Figure 3:**
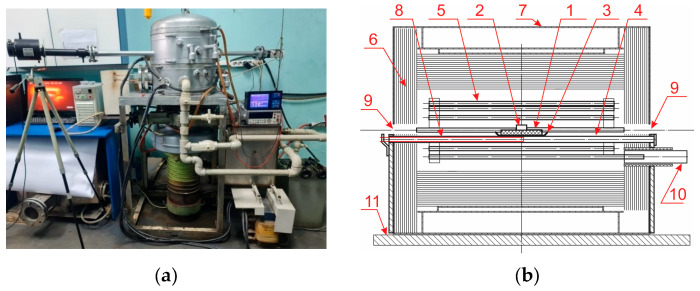
Unit intended to determine the wetting contact angle under high vacuum conditions: (**a**) Photo of the unit; (**b**) Furnace diagram. 1—ceramic substrate ∅40 mm; 2—titanium cylinder ∅10 × 4 mm; 3—molybdenum boat; 4—tungsten supports; 5—molybdenum heating elements; 6—screens made of molybdenum and heat-resistant steel; 7—outer casing of the furnace; 8—tungsten-rhenium thermocouple; 9—observation windows; 10—current leads; 11—adjustable table.

An experimental unit has been made to determine the parameters of wetting ceramic substrates with titanium melt (Figure 3a). This unit provides heating of a ceramic substrate with a titanium alloy cylinder installed on its surface to a specified temperature, video recording of the process of spreading titanium melt on the surface of a ceramic sample in a horizontal projection. The heating process takes place under high vacuum conditions provided with a two-stage vacuum pumping system. During heating, the melt temperature is recorded using Thermoscope-800-2C-VT1 (Yekaterinburg, Russia), a stationary infrared pyrometer of spectral ratio.

**Figure 4:**
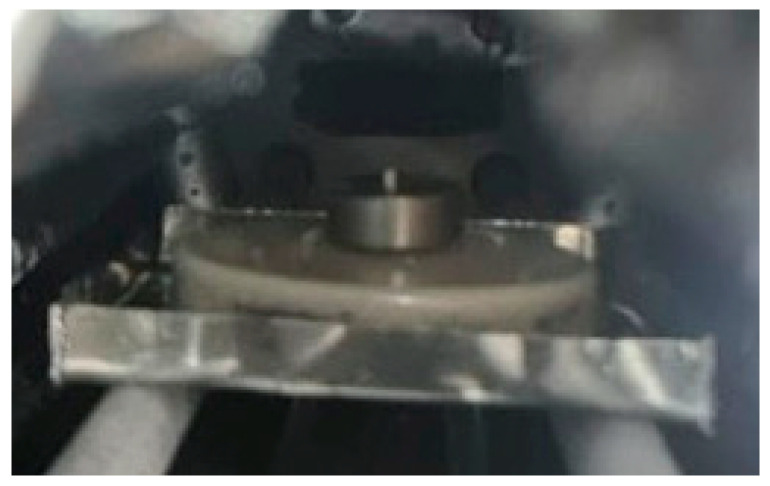
The appearance of a CaTiO_3_ substrate with a titanium sample installed in the furnace chamber.

A cylinder made of titanium alloy of the Grade 2, ∅10 mm and 5 mm high was installed in the center of the CaTiO_3_ substrate (Figure 4).

The sample on a molybdenum pallet was then placed in the unit furnace to determine the wetting contact angle (Figure 3b). When a residual pressure of 1–2 × 10^−4^ mmHg was reached in the unit chamber the furnace was heated. A video recording was turned on at the beginning of titanium melting while the melt temperature was continuously recorded. When the specified temperature was reached, isothermal holding was conducted. At the end of the holding, the heating of the furnace was stopped, and the sample was cooled under conditions of continuous pumping to 50–100 °C.

Table 4 shows the experimental conditions.

When the wetting contact angle was measured, freeze frames of the video of the melt spreading process on the substrate were used. Measurements of the wetting contact angle were conducted using the ImageJ 1.53q program.

The structure of the contact zone between the melt and the ceramic substrate and the composition of the phases formed within it were studied using a Leica DM IRM inverted optical microscope (Leica Microsystems GmbH, Wetzlar, Germany) and a JEOL JXA-8230 electron probe microanalyzer (JEOL, Tokyo, Japan) in COMPO (compositional contrast) mode. The microstructure was examined with an optical microscope before and after etching. The titanium structure was etched by wiping the surface of the section with a cotton swab soaked in a solution of hydrofluoric and nitric acid. The elemental composition of the phases was studied using the EDS (energy dispersive spectrometry) method at a current of 20 nA and a voltage of 20 kV. These studies were performed on transverse sections of the obtained samples. The sections were prepared using traditional technology. After cutting on a diamond disk, the surface of the samples was ground using SiC abrasive paper and then polished using diamond pastes. After each grinding and polishing cycle, the abrasive material’s dispersion was increased. The phase composition of the substance released from the titanium droplet was analyzed using a D8 Advance X-ray diffractometer (BRUKER) with α-Cu radiation (λ~1.54 A).

## 3. Results

### 3.1. Features of the Process of Wetting CaTiO_3_ Substrates with Different Structures with Titanium

**Figure 5:**
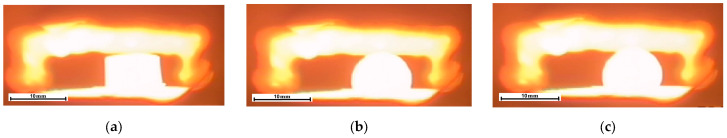

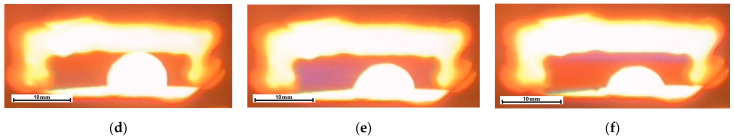
Changes in the shape of a droplet’s surface on a CaTiO_3_ substrate (from 0 to 0.2 mm powder) under continuous temperature increase in a furnace at a rate of 0.33 ± 0.05 °C/s. (**a**)—1610 °C; (**b**)—1668 °C; (**c**)—1678 °C; (**d**)—1708 °C; (**e**)—1718 °C; (**f**)—1728 °C.

Figure 5 shows the key stages of droplet shape change during interaction between titanium melt and the surface of a calcium titanate substrate with increasing temperature from the melting point to 1760 °C. From the melting point to 1740 °C, molten titanium forms a droplet of a regular hemispherical shape. At temperatures above 1718 °C, oscillations of the melt surface with periodic splashes and the ejection of microdroplets were observed. This indicates gas evolution at these temperatures. This process was most noticeable on substrates obtained from powders smaller than 0.1 mm.

**Figure 6:**
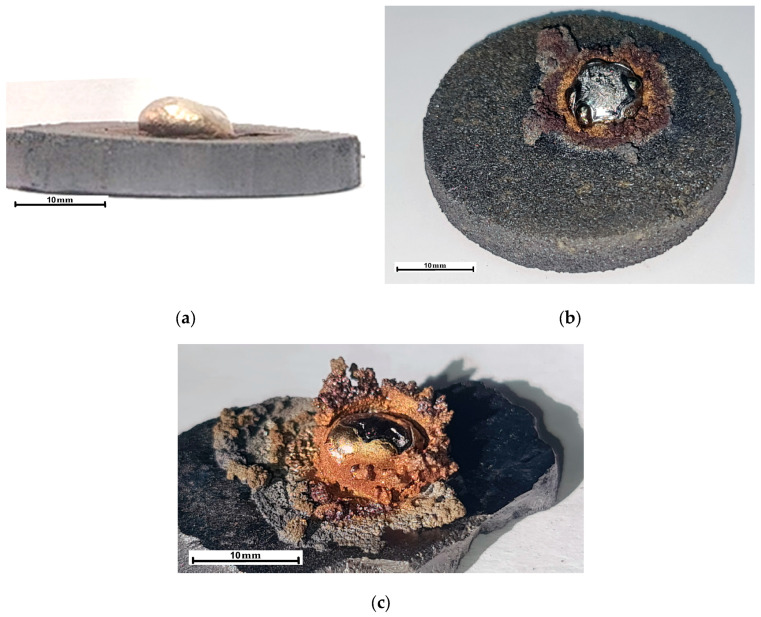
The influence of the CaTiO_3_ substrate characteristics on the formation of a TiO rim around a droplet of molten titanium during their interaction under heating at 1720 °C for 230 s. (**a**)—≤0.1 mm; (**b**)—0.1–0.2 mm. 0.2–0.3 mm. 0.3–0.5 mm; (**c**)—hard alloy substrate.

Of particular scientific interest is the release of a substance in a solid–liquid state from under a drop. This substance formed growths around the droplet’s perimeter with a loose, heterogeneous structure, partly reminiscent of dendritic crystals (Figure 6). This rim around the melt droplet formed upon heating above 1700 °C and, in some cases, prevented observation of the contact angle, leading to the termination of the experiment. The intensity of this phenomenon increased with increasing particle size of the CaTiO_3_ powders used to obtain the substrate. Thus, in the case of a substrate made of powders smaller than 0.1 mm, a rim barely protruding above the surface formed around the droplet (Figure 6a). For substrates made of powders of 0.1–0.2 mm, 0.2–0.3 mm, and 0.3–0.5 mm, the intensity of rim formation increased (Figure 6b). This phenomenon was most significantly observed when a substrate made of fused CaTiO_3_ was used (Figure 6c).

X-ray phase analysis of the material taken from the surface of sample No. 5 revealed that it was composed of calcium titanate (Table 5). However, microstructural examination revealed dispersed TiO inclusions uniformly distributed within the calcium titanate matrix.

**Figure 7:**
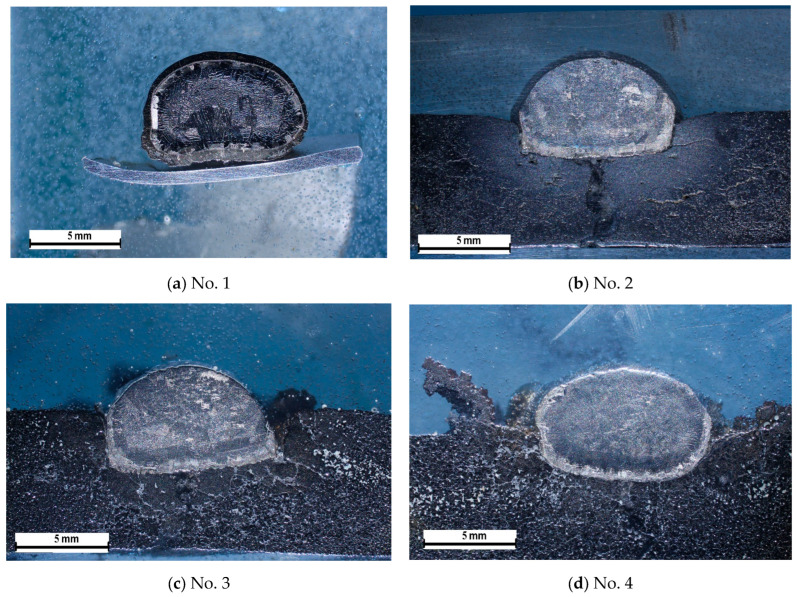

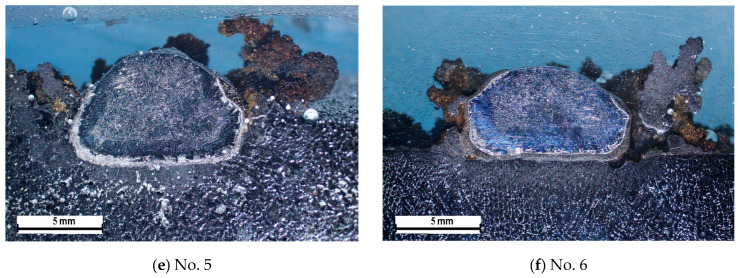
Macrostructure of the sample in cross-section. Sample obtained on a CaTiO_3_ substrate with (**a**) fraction of 0.063 mm green substrate; (**b**) ≤0.1 mm; (**c**) 0.1–0.2 mm; (**d**) 0.2–0.3 mm; (**e**) 0.3–0.5 mm; (**f**) sample on a CaTiO_3_ alloy—0.063 mm sintered.

Figure 7 shows macrographs of cross-sections of the obtained samples. Analysis of these images indicates that, during interaction with the substrate, the titanium melt droplet sinks below the substrate level. Sample #6, which used a cast substrate, is least susceptible to this phenomenon.

When examining the macrostructure of sample #1, obtained using a green substrate, a continuous yellow shell formed on the surface of the titanium droplet (Figure 7a). In other cases (Figure 7b–f), a yellow layer formed at the melt-ceramic interface and on the lateral surfaces of the droplet, but no formation was detected on the surface. The exception was sample No. 6, in which such a layer was detected on the surface of the droplet’s hemisphere.

### 3.2. Results of Measuring the Contact Angle of Wetting of CaTiO_3_ Substrates with Titanium Melt

**Figure 8:**
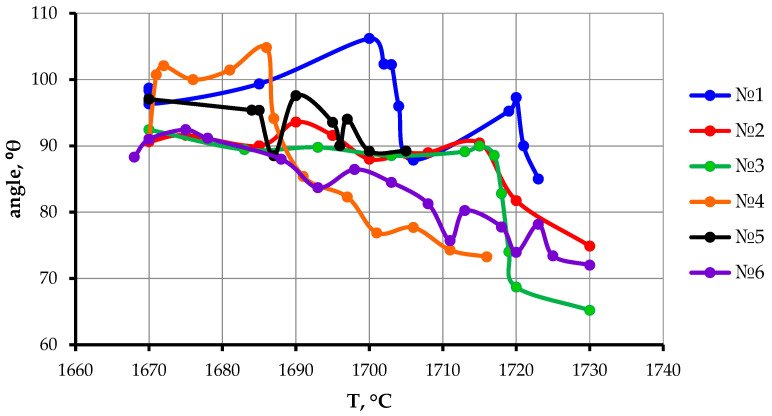
Temperature dependence of the contact angle of wetting of titanium melt on CaTiO_3_ substrates with different characteristics. No. 1—0.063 mm green substrate, No. 2—0.1 mm, No. 3—0.2 mm, No. 4—0.3 mm, No. 5—0.5 mm, No. 6—0.063 mm sintered.

Figure 8 shows the temperature dependence of the contact angle of the titanium melt on CaTiO_3_ substrates. As can be seen from the obtained curves, with increasing temperature, the contact angle initially increases above 90°, stabilizes or increases slightly, and then gradually decreases. This decrease in contact angle is clearly due to the melt droplet sinking below the substrate surface. Since the contact angle measurement was performed relative to the substrate surface, such a measurement did not provide an objective result. The discontinuities in the contact angle curves are due to periodic gas release, which was observed visually. This resulted in a change in the shape of the melt droplet’s surface.

Analysis of the curves shown in Figure 8 indicates that near the melting point of titanium, the contact angle exceeds 90°. This indicates that at these temperatures, the interaction of the titanium melt with CaTiO_3_ is minimal, and the penetration of titanium droplets into the CaTiO_3_ substrate is insignificant. Moreover, the dispersion of the powders used to manufacture the substrate has a significant effect on the contact angle. Substrates obtained from powders with large fractions of 0.2–0.3 mm and 0.3–0.5 mm are wetted in the range of 1670–1685 °C with a contact angle of 90–104°. Substrates obtained from more dispersed powders are wetted by the titanium melt (<0.1 mm and from 0.1 to 0.2 mm) at smaller contact angles (90–93°), while the plateau region expands to 1717 °C. Substrates made of sintered CaTiO_3_ powders with a dispersion of <0.063 mm are wetted by the melt at a contact angle of 88–92° from the moment of titanium melting and up to 1682 °C. In this case, the wetting of the sintered substrate and the substrate made of powders with similar dispersion (<0.063 mm), but in the unpressed and unsintered state, are radically different. For a green substrate, from the moment titanium melts to 1700 °C, the contact angle increases from 95° to 106°, then decreases to 88° (at 1707 °C), and then gradually increases again to 97° at 1720 °C, after which it decreases. This indicates that capillary effects do not influence the wetting of CaTiO_3_ substrates by titanium. However, the combination of substrate properties, such as particle size and porosity, determines its surface energy.

Since a study of the temperature dependence of the contact angle of CaTiO_3_ substrates revealed that at temperatures close to the melting point of titanium, the contact angle is close to 90° and even exceeds this value, this indicates that wetting does not develop. However, since the studies were conducted under conditions where the temperature gradually increased, the contact angle cannot be considered steady-state. To assess the change in the contact angle over time, tests were conducted under conditions close to isothermal. Sintered substrates of 0.063 mm powders were used for these tests, which was due to the complete absence of rim formation, which interferes with contact angle measurements. Three temperatures were chosen: 1690, 1705 and 1720 °C. The choice of these temperatures is justified by the fact that when studying the temperature dependence (Figure 8, curve 6), at these temperatures both stabilization and a decrease in the contact angle were observed.

**Figure 9:**
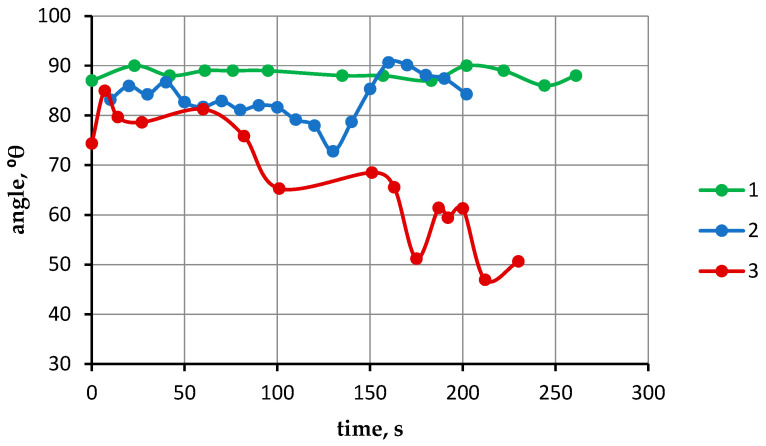
Dependence of the contact angle θ on time: 1—1690 °C; 2—1705 °C; 3—1720 °C.

The results of these tests showed that the nature of the change in the contact angle under isothermal conditions varies depending on the temperature (Figure 9). Isothermal holding at 1690 °C does not cause a change in the contact angle from the moment of melting until the end of the experiment (Figure 9, line 1). Under isothermal holding conditions at 1705 °C (Figure 9, line 2), the contact angle fluctuates between 83° and 86° during the first seconds and then stabilizes at 82°. Then, at 100 s, a gradual decrease in the wetting angle occurs from 82° to 72°. Then, at 140 s, the angle increases from 72° to 90°. Further holding leads to a decrease in the contact angle from 90 to 84°. This is explained by the fact that over time, as a result of reactive diffusion interaction, an intermediate phase is formed at the substrate/melt contact boundary, the wetting of which by the titanium melt may differ from the wetting of the CaTiO_3_ surface. The surface topography of the forming intermediate phase layer can also affect wetting parameters. However, despite fluctuations in the contact angle over time, its average value remains close to 84°.

The dependence of the contact angle of a CaTiO_3_ substrate on the contact time by the titanium melt at 1720 °C is shown in Figure 9 (line 3). As can be seen from the data, under isothermal holding conditions, the contact angle initially increases abruptly to 84°, after which it stabilizes at 81–79° over 50 s and then decreases to 47° with periodic fluctuations. This change in the contact angle indicates an intensification of the reaction after stabilization at the initial stage, leading to gradual penetration of the titanium melt droplet below the substrate surface. The abrupt changes in the contact angle are caused by the release of a gas phase and the resulting oscillation of the droplet surface.

Thus, near the melting point, the titanium melt poorly wets CaTiO_3_ substrates. As the temperature rises above the melting point, the contact angle stabilizes at a certain level, depending on the particle size of the substrate powders and their porosity. As the temperature rises above a certain level, the interaction between the substrate and the titanium melt intensifies, causing a drop of melt to descend below the substrate surface. This gives a false result when measuring the contact angle, indicating a decrease. Under isothermal conditions of melt-substrate interaction at 1705 °C, the contact angle stabilizes at an average of 84°, despite fluctuations in value. At 1720 °C, after stabilizing for the first 50 s, the contact angle decreases with further exposure, which is also associated with increased reactive interaction.

### 3.3. Study of Microstructure and Phase Composition

The structure and composition of the phases formed at the CaTiO_3_/Ti contact interface and in the bulk of titanium were investigated in all samples obtained during the wetting parameter studies. It was found that the structure was similar in all cases.

**Figure 10:**
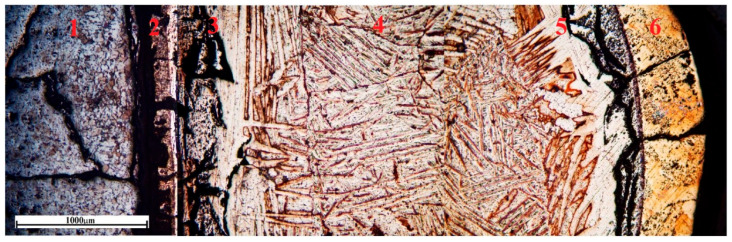
Change in the microstructure across the cross-section of a CaTiO_3_/VT1-0 alloy sample after isothermal interaction at 1720 °C for 430 s.

This article presents images of the structure of a sample obtained after isothermal holding at 1720 °C for 430 s. Microstructure analysis and microprobe analysis of the sample cross-section revealed characteristic zones indicating the development of reaction interaction and diffusion of reaction products into the melt volume (Figure 10): 1—CaTiO_3_ ceramics impregnated with titanium melt; 2—two two-phase layers of CaTiO_3_ + αTi(O) (not formed in samples obtained without isothermal holding), 3—layer of solid solution of oxygen in titanium; 4—practically pure titanium; 5—layer of solid solution of oxygen in titanium; 6—titanium oxide TiO with a polyhedral fine-grained structure.

**Figure 11:**
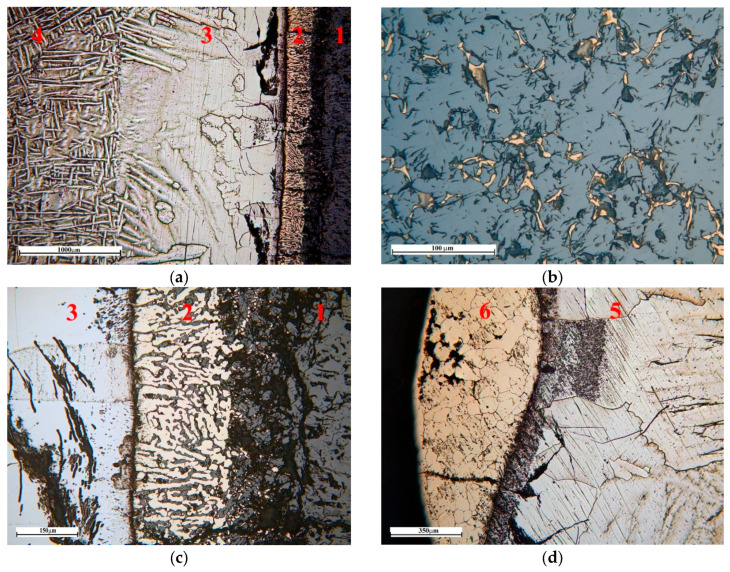
Typical microstructure in individual zones across the cross-section of samples obtained under isothermal holding conditions of CaTiO_3_/Ti. (**a**)—zones 1–4 (×50); (**b**)—zone 1 (×500); (**c**)—zones 1–3 (×200); (**d**)—zone 4 (×100), 5—solid solution of oxygen in titanium, 6—TiO.

In zone No.1, veins of a titanium phase with a high oxygen content were detected along the CaTiO_3_ grain boundaries (Figure 11b). The penetration depth from the ceramic substrate surface in some cases reaches 1.2–1.3 mm. This process presumably develops due to the formation of a liquid phase along the CaTiO_3_ grain boundaries due to reaction with the titanium melt.

**Figure 12:**
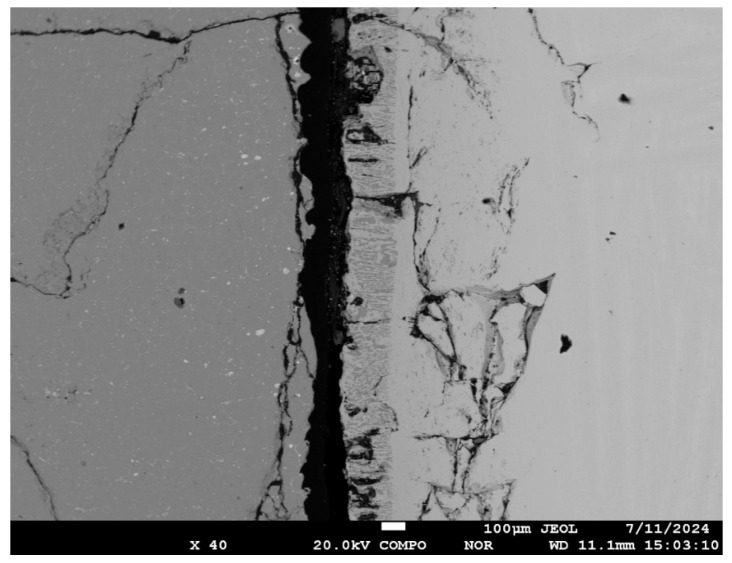
The microstructure of the CaTiO_3_/Grade 2 alloy reaction zone displayed using an electron microscope.

**Figure 13:**
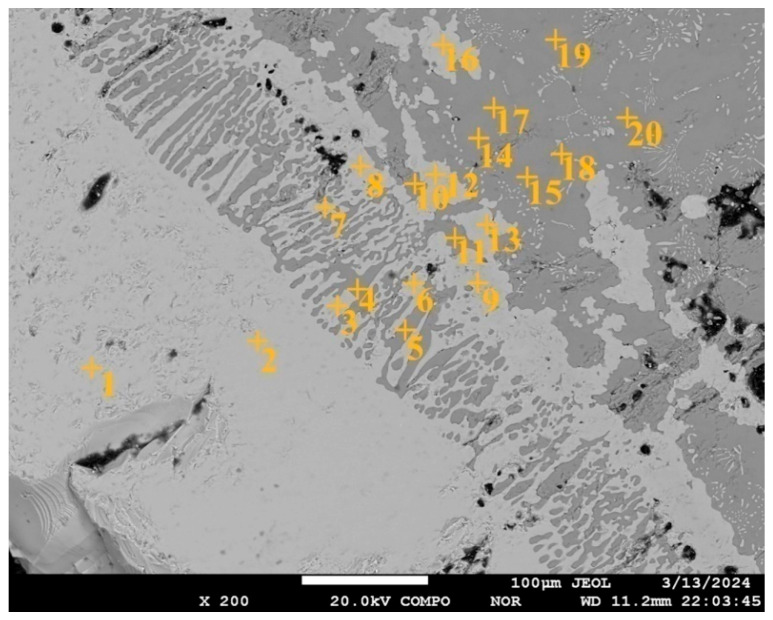
The microstructure of the reaction zone from the titanium side at the contact boundary with the ceramic substrate, indicating the locations of microprobe analysis (Table 6).

Figure 12 shows a typical microstructure formed at the interface between the titanium melt and the CaTiO_3_ substrate. Two layers, each up to 220 μm wide, form directly at the interface between the melt and the ceramic substrate (Figure 11a,c). These layers are formed by a two-phase mixture. The layer closest to CaTiO_3_ is formed by globular and polyhedral precipitates of a solid solution of oxygen and calcium in titanium (points 16, 12, 13 in Figure 13 and Table 6), which are distributed in a CaTiO_3_ matrix (points 14, 15, 17, 18, 19 and 20 in Figure 13 and Table 6). The size of globular inclusions of a titanium-based solid solution is on average 3 μm, and polyhedral ones are up to 10 μm (Figure 13). This structure suggests the formation of a layer of a liquid or solid–liquid phase containing Ca, Ti and oxygen as a result of the reaction between the titanium melt and CaTiO_3_. Moreover, after crystallization, the composition of the oxide phase in all areas corresponds to the composition of the original CaTiO_3_.

A two-phase region formed in the second layer, consisting of a mixture of columnar, globular, and droplet-shaped crystals in the titanium matrix (Figure 11c and Figure 13). The width of this zone is 90–130 μm. The columnar crystals are oriented predominantly perpendicular to the substrate surface. Microprobe analysis of the transition zone indicates that it is formed by a two-phase mixture of a solid solution of oxygen and calcium in titanium (points 3, 6, 7, 8, 9 in Figure 13 and Table 6) and columnar oxide precipitates. The composition of the oxide phase is close to CaTiO_3_, but with a slightly higher oxygen content (points 4, 5, and 10 in Figure 13 and Table 6). The rounded shape and the presence of globular inclusions of a titanium-based solid solution in their structure indicate that the oxide phase material was in a liquid state. This structure may indicate eutectic decomposition of the melt during cooling.

Near the contact boundary between the titanium and the substrate, pores of various shapes and sizes (10–20 µm) were formed.

**Figure 14:**
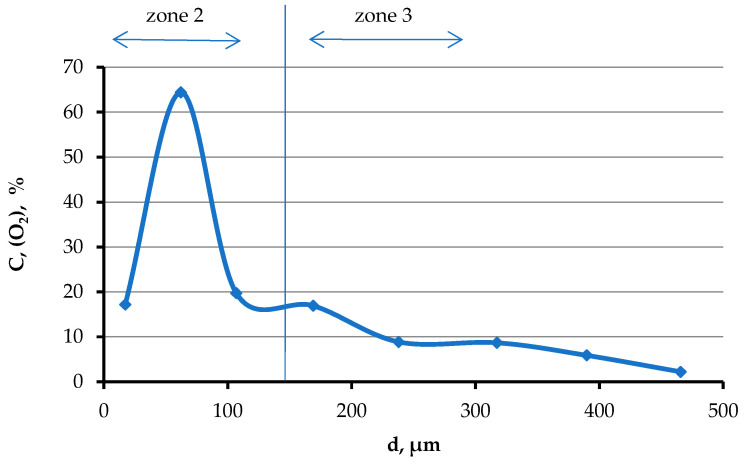
Change in oxygen concentration as the titanium melt moves away from the contact boundary of the titanium melt with the substrate.

Titanium has a structure characteristic of α titanium in zone No. 3 (Figure 11a). This area has a coarse-grained dendritic structure. The width of this layer is up to 1.5 mm. Titanium contains a high concentration of oxygen in zone No. 3 immediately in the vicinity of zone No. 2 (points 1 and 2 in Figure 13 and Table 6), while calcium is not found in these points. With increasing distance from the boundary with zone No. 2, the oxygen concentration decreases from ~16 to 2 mol.% already at a distance of ~400 μm (Figure 14). Further, oxygen is not detected by microprobe analysis.

**Figure 15:**
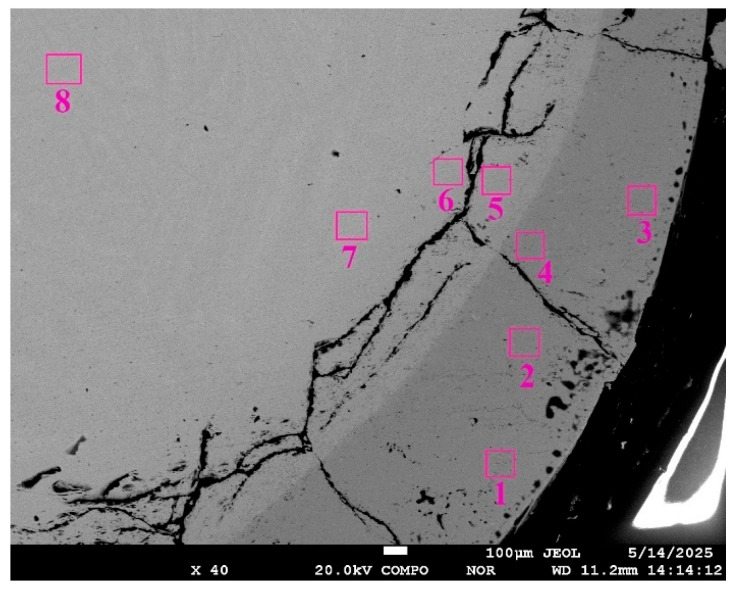
The locations of microprobe analysis of the composition in zones No. 3 and No. 4.

The titanium structure is acicular in zone No.4. Microprobe analysis of a cross-section of this zone revealed no oxygen or calcium content (points 6, 7, and 8 in Figure 15).

As we approach zone No. 6 at a distance of ≤300 µm, the oxygen concentration in zone No. 5 increases (Figure 15, Table 7, point 5) and directly at the border with zone No. 6 reaches 24.6 mol%.

Zone No. 6 is a layer that forms a fragmented shell of the titanium melt droplet dome, which is adjacent to the furnace atmosphere at a residual pressure of 1–2 × 10^−4^ mmHg. The microstructure of the metal in zone No. 6 is fine-grained and acquires a light-brown golden color upon etching (Figure 11d); before etching, the surface had a metallic luster. Analysis of the composition along the cross-section of this zone revealed a ratio of atoms close to the equiatomic ratio (Figure 13, Table 7, points 1, 2, 3, and 4). It indicates that the material forming this layer is a TiO compound. At the boundary with zone No.3, the oxygen concentration in it decreases to 27.6 mol%

## 4. Discussion

The thermodynamic calculations presented at the beginning of this article, which indicate that calcium metatitanate should not interact with titanium melt, are inconsistent with the experimental results. Similar observations have been made by other researchers [35,36,37]. Therefore, predicting such an interaction cannot be based solely on the Gibbs free energy, as the solubility and activity of the refractory compound in the titanium melt play a significant role.

In the case of reactive interaction, substrate wetting by the melt typically increases. In the titanium melt/CaTiO_3_ substrate system, the contact angle is large, which could indicate insignificant diffusion and reaction interaction. However, studies of the melt-substrate contact zone revealed that the titanium melt reacts with calcium titanate. The following features of the reaction between CaTiO_3_ and the titanium melt are noted:

- During the interaction process, due to capillary wetting, the titanium melt penetrates the substrate, where it interacts with CaTiO_3_ and becomes saturated with oxygen.
- Calcium, oxygen, and titanium diffuse from the substrate surface into the melt due to substrate dissolution.
- Two two-phase layers are formed at the CaTiO_3_/Ti interface, consisting of a mixture of CaTiO_3_ and a solid solution of oxygen and calcium in titanium. One layer is formed by oriented CaTiO_3_ crystals alternating with crystals of a solid solution of oxygen and calcium in titanium.
- A liquid phase forms near the interface in the ceramic substrate.
- Despite a relatively long isothermal holding, calcium and oxygen penetrated into the titanium to a small depth (up to 90–130 µm and up to ±400 µm, respectively) from the interface with the CaTiO_3_ substrate. A coarse-grained α-Ti layer with variable oxygen content formed.
- The central region of the titanium droplet is composed of titanium with low impurity levels.
- A high-oxygen shell, similar in composition to TiO, formed on the surface of the titanium droplet, bordering a narrow zone of α-Ti with increased oxygen content.

The observed changes in composition and structure in the titanium melt/CaTiO_3_ substrate diffusion pair indicate the development of complex physicochemical processes at the interface. Since the interaction between the melt and the substrate is dictated by the system’s tendency to reach equilibrium, some of the observed phenomena can be described by examining regions of the Ti-Ca-O system’s phase diagram.

**Figure 16:**
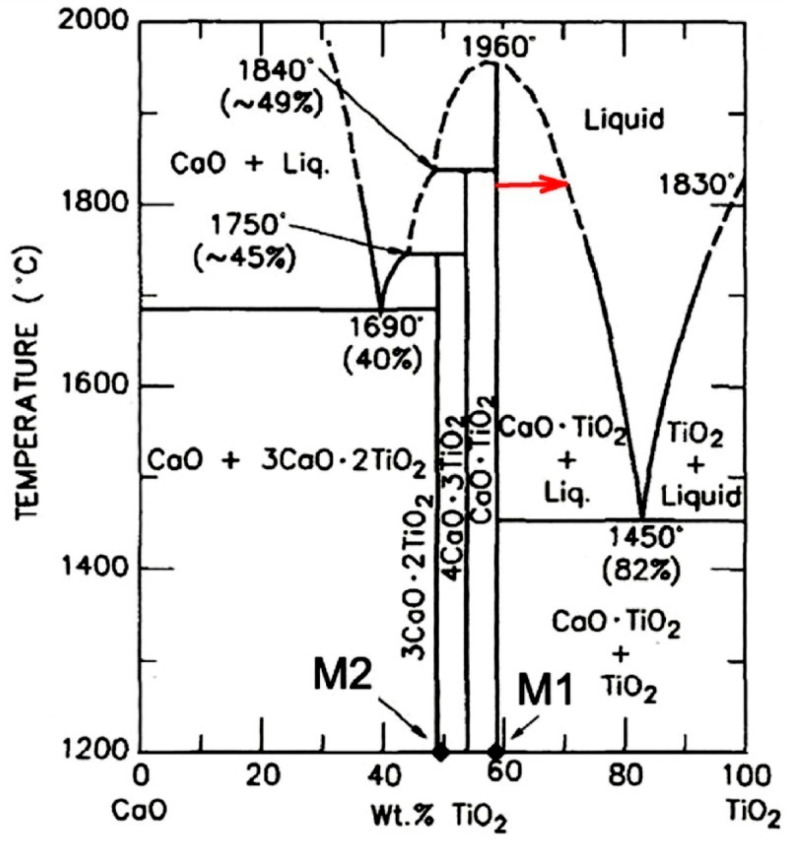
Phase diagram of CaO–TiO_2_ system reprinted from ref. [40].

According to the CaO-TiO_2_ phase diagram (Figure 16), with a decrease in the CaO concentration (the concentration change is shown by the red arrow in Figure 16), a liquid phase is formed due to interaction with the titanium melt. This phase crystallizes upon cooling with the precipitation of primary CaTiO_3_ crystals and then, via a eutectic reaction, with the precipitation of a CaTiO_3_ + TiO_2_ mixture at 1450 °C. In the experiments conducted, the formation of two layers of a mixture of CaTiO_3_ + αTiO, Ca crystals was revealed in the transition layer (zone No. 2). Since the CaTiO_3_-Ti phase diagram has not been constructed, it can be concluded by analogy with the CaO-TiO_2_ diagram that, due to a decrease in the CaO proportion, a liquid phase is formed at the contact boundary with the titanium melt, which crystallizes as a eutectic. This is confirmed by the results of studies of the substrate microstructure. The only possible cause for the decrease in CaO content at the interface with the titanium melt is the direct reduction of calcium from the CaTiO_3_ compound by titanium (reaction 2, Figure 1) and its subsequent evaporation. According to the Ca-Ti phase diagram [38,39], calcium does not form solid solutions with titanium, but does not dissolve significantly in liquid titanium. This explains why calcium is found in titanium only in the reaction zone, in an area where, in addition to calcium, the composition contains a high concentration of oxygen (up to 25 at.%).

It is known [41] that the vapor pressure of metallic calcium increases significantly with increasing temperature and decreasing atmospheric pressure. The boiling point of calcium at atmospheric pressure is 1484 °C. Obviously, under the experimental conditions (1720 °C, 1–2 × 10^−4^ mmHg), the reduction of calcium to the metallic state led to its release in the form of a vapor phase. It is known that in a vacuum at elevated temperatures, the predominant vapor phase above the surface of CaTiO_3_ is calcium vapor, TiO, TiO_2_ [42]. However, the vapor pressure in the absence of a reducing agent is extremely small. Thus, in the temperature range of 1680–1720 °C, the vapor pressure of these substances is: for Ca 4.54–8.89 × 10^−8^ mmHg, for TiO 0.8–1.8 × 10^−8^, for TiO_2_ 2.4–5.01 × 10^−8^ mmHg. At these temperatures, dissociation of titanium oxides in vacuum does not occur. In this regard, under the experimental conditions considered, during the reaction of the titanium melt with CaTiO_3_, calcium vapor will be the predominant vapor phase. The proportion of TiO will increase. However, the mechanism for removing calcium vapor is not fully described by the observed structure of the transition zone, nor by the observed spreading of a titanium droplet over the surface of the CaTiO_3_ plate. Thus, isolated bursts (oscillations) caused by the release of gas microbubbles, as well as micropores in the transition zone, do not allow us to consider these processes of calcium vapor release as the main pathway for its removal from the reaction zone. The gap in the transition zone observed in the initial stages of the study was mistakenly taken for a gas gap between the melt and the substrate. However, adjustments to the sample preparation process for the thin sections revealed that the gap formed as a result of cracking and destruction of one of the two-phase layers. Calcium released during the reaction is known to be almost insoluble in the titanium melt [37,39]. This suggests that the calcium released during the interaction diffuses in the oxygen-enriched phase formed in the transition zone and is removed, including directly from the droplet surface, without mixing with the titanium melt. The significant non-uniformity of oxygen distribution across the cross-section of the titanium melt droplet can be explained by the formation of two stratified liquids. According to the Ti-O phase diagram, a monotectic transformation occurs in the system at atmospheric pressure in the oxygen concentration range of 37–53 at.% above 1780 °C. A decrease in pressure likely causes a change in equilibrium in the Ti-O system, and as a result, the phase diagram transforms with decreasing temperatures of the phase transformation lines. This may explain the formation of a TiO shell on the surface of a titanium droplet at 1720 °C and at higher temperatures, and the retention of the droplet in a liquid state during the experiment, as was visually observed.

According to the Ti-O phase diagram [43], the melting point of titanium increases with increasing oxygen concentration. Consequently, in the case of isothermal interaction of titanium with CaTiO_3_ in the temperature range of 1670–1730 °C, βTi and αTi layers should form at a certain distance from the contact boundary with CaTiO_3_. And above 1720 °C, only the αTi layer. The αTi(O) layer was revealed during microstructural studies in the transition zone (Figure 11c). According to the Ti-O phase diagram, at 1720 °C, the dissolution of oxygen in the titanium melt should be at the level of 5–6 at.%. However, during the study of zone No. 4, corresponding to the melt, oxygen was not detected by microprobe analysis. It is likely that during isothermal holding, oxygen diffuses toward the outer shell of the droplet, initially forming a thin layer of the αTi(O) solid solution identified in the studies, and then bonding to form TiO.

The large contact angle between the titanium melt and the CaTiO_3_ substrate, despite the development of the reaction, is explained by the formation of both intermediate phases at the CaTiO_3_/Ti interface, including a liquid layer, and presumably a subatomic Ca layer. Clearly, the formation of a liquid TiO layer near the surface, by altering the surface tension, can also alter the surface tension of the melt, influencing the wetting process.

**Figure 17:**
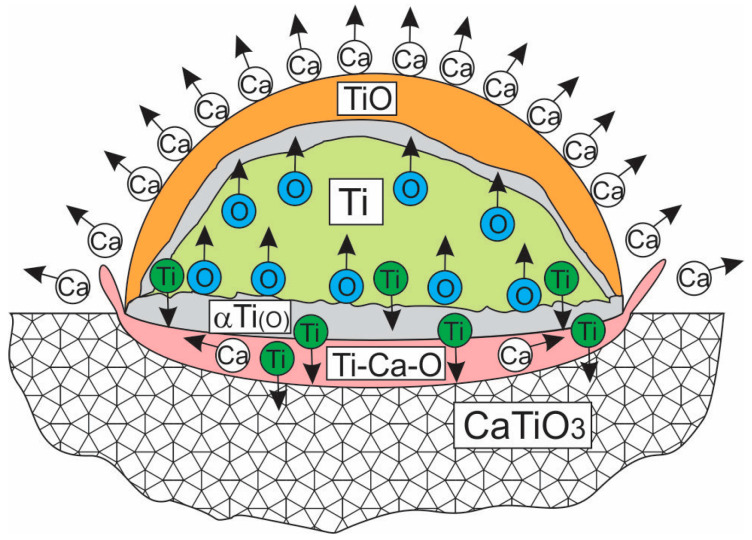
A schematic representation of the reaction processes of CaTiO_3_ with titanium melt.

Thus, the mechanism of interaction between the titanium melt and the CaTiO_3_ substrate can be described in terms of the gradual dissolution of CaTiO_3_ in the melt with the simultaneous reduction of metallic calcium according to reaction Equation (2) and the redistribution of oxygen and titanium within the melt. The formation of a subatomic calcium layer, an αTi(O) solid solution layer, in the contact zone limits the reactive interaction and prevents the ceramic from being wetted by the melt. As the temperature increases above 1720 °C, the αTi(O) solid solution layer becomes structurally non-uniform, causing a sharp increase in the reactive interaction. However, the mechanism by which the powder size of the CaTiO_3_ substrates influences the contact angle and the threshold temperature at which the reactive interaction begins remains unclear. The processes occurring during the interaction between CaTiO_3_ and the titanium melt are shown schematically in Figure 17.

Near the melting point of titanium, CaTiO_3_ is not significantly susceptible to the aggressive effects of the titanium melt compared to previously studied materials [7]. Since calcium vapor release during interaction with calcium titanate is more moderate than that of BaZrO_3_ and SrZrO_3_, the removal of its vapor from the contact zone is more stable. This significantly protects the titanium melt from further contamination.

These properties of CaTiO_3_ ceramics make it a promising material as a base for molding mixtures for casting titanium alloys. Under short-term contact conditions, the titanium melt will be insignificantly saturated with oxygen, which will not significantly affect the mechanical properties of the castings. Pure CaTiO_3_ cannot be used as a refractory crucible material for melting titanium alloys in vacuum induction furnaces, as long contact times with the melt and melt circulation will lead to significant oxygen contamination. Further research into modifying this compound will likely improve its performance for this application.

## 5. Conclusions

This study presents the following conclusions:

- Calcium titanate CaTiO_3_, obtained by melting and subsequent sintering, is poorly wetted by titanium melt at low temperatures and short contact times. As the temperature increases above a threshold value, the reactive interaction between CaTiO_3_ and the titanium melt intensifies significantly. The wetting parameters and the critical temperature for the transition to active interaction depend on the size of powders of the powders used to produce sintered CaTiO_3_ ceramics. The optimal size of powders is 0.1–0.2 mm.
- The interaction of titanium melt with a CaTiO_3_ substrate includes the following processes: impregnation of the ceramic material with titanium melt; reaction with the formation of a Ca-Ti-O melt layer of variable composition and the reduction of calcium to a metallic state, followed by its diffusion from the transition zone with evaporation; Oxygen diffusion into the titanium melt, forming a layer of αTi(O) solid solution, and subsequent diffusion of oxygen atoms to the outer surface of the droplet, forming a TiO layer.
- The observed phase formation and oxygen distribution in the reaction zone cannot be fully explained by the phase diagrams of the CaO-TiO_2_ and Ti-O systems. It is assumed that the high-temperature portion of the Ti-O phase diagram is transformed at low pressure, decreasing the melting point of TiO and forming a monotectic;
- The obtained data allow us to recommend CaTiO_3_ as a filler for mold materials used in casting titanium alloys.

## Figures and Tables

**Table 1:** A comparison of the physical properties of CaTiO_3_ with materials most promising for refractories and molding materials for casting titanium alloys.

Material	Density, ρ, g/cm^3^	Thermal Conductivity, W/(m·K)	Heat Capacity, cal/(mol·K)	Melting Point, °C
CaTiO_3_	3.98–4.1	2.2–2.4	23.9	1975
CaZrO_3_	4.78–4.95	~2.7	23.88	2350
Y_2_O_3_	5.0	8.5	22.94	2400

**Table 2:** Chemical composition of Grade 2, %.

Ti	Fe	C	Si	N	O	H
99.24–99.7	0.25 max	0.07 max	0.1 max	0.04 max	0.2 max	0.01 max

**Table 3:** Apparent density and porosity of substrates obtained from CaTiO_3_ powders of different fractions.

Sample No.	Fraction Size	*p*_app_	P_open,%
1	<0.063 mm (not pressed)	1.35	67
2	≤0.1 mm	2.65	23.86
3	0.1–0.2 mm	2.42	38.00
4	0.2–0.3 mm	2.49	37.24
5	0.3–0.5 mm	2.46	37.74
6	0.063 pressed	2.75	19.54
7	monolithic	4.04	0.1

**Table 4:** Experimental conditions.

Sample No.	The Particle Size of Calcium Titanate Powders	Interaction Duration, s	Maximum Interaction Temperature, °C
1	<0.063 mm (not pressed)	323	1723
2	≤0.1 mm	278	1737
3	0.1–0.2 mm (+initial)	240	1730
4	0.2–0.3 mm	120	1716
5	0.3–0.5 mm	98	1705
6	0.063 pressed	240	1730
7	Hard alloy substrate	100	1730

**Table 5:** Phase composition of the rim substance from the surface of sample No. 5.

Pattern No.	Compound Name	Formula	S-Q
PDF 00-022-0153	Perovskite, syn	CaTiO_3_	100.0%

**Table 6:** The results of microprobe analysis of the transition zone at the points specified in Figure 11.

No.	Ti	Ca	O
	mol%	mol%	mol%
1	89		11
2	84		16
3	80	1	19
4	16	17	66
5	16	17	68
6	75	1	24
7	83	2	15
8	74	0	26
9	78	0	22
10	17	17	65
11	16	27	66
12	81	1	18
13	82	1	17
14	17	17	65
15	17	17	66
16	79	1	20
17	17	17	65
18	17	17	66
19	17	18	65
20	17	17	65

**Table 7:** The results of microprobe analysis of the transition zone in the areas specified in Figure 13.

No.	Ti	O
	mol%	mol%
1	48	52
2	49	51
3	49	51
4	50	50
5	89	11
6	100.0	nd
7	100.0	nd
8	100.0	nd

“nd” stands for “not detected”.

## Data Availability

The original contributions presented in this study are included in the article. Further inquiries can be directed to the corresponding author.

## References

[1] Panichkin A.V., Uskenbayeva A.M., Imanbayeva A., Temirgaliyev S., Djumabekov D. (2016). Interaction of titanium melts with various refractory compounds. Complex Use Miner. Resour..

[2] Fashu S., Lototskyy M., Davids M., Pickering L., Linkov V., Tai S., Renheng T., Fangming X., Fursikov P., Tarasov B. (2020). A review on crucibles for induction melting of titanium alloys. Mater. Des..

[3] Duan B., Chen G., Xiong F., Liu X., Zhang X., Feng Q., Lan B., Xiao Y., He S., Mao L. (2020). A Review on the Preparation Techniques of Titanium Alloy and the Selection of Refractories. J. Mater. Metall..

[4] Frueh C., Poirier D.R., Maguire M.C., Harding R.A. (1996). Attempts to develop a ceramic mould for titanium casting—A review. Int. J. Cast Met. Res..

[5] Kartavykh A.V., Tcherdyntsev V.V., Zollinger J. (2009). TiAl–Nb melt interaction with AlN refractory crucibles. Mater. Chem. Phys..

[6] Frenzel J., Zhang Z., Neuking K., Eggeler G. (2004). High quality vacuum induction melting of small quantities of NiTi shape memory alloys in graphite crucibles. J. Alloys Compd..

[7] Kenzhaliyev B., Panichkin A., Uskenbayeva A., Chukmanova M., Mamaeva A., Kshibekova B., Alibekov Z. (2024). Interaction of liquid titanium with zirconates and titanates of some alkaline earth metals. Mater. Res. Express.

[8] Li C.H., Gao Y.H., Lu X.G., Ding W.Z., Ren Z.M., Deng K. (2010). Interaction between the ceramic CaZrO_3_ and the melt of titanium alloys. Adv. Sci. Technol..

[9] Klotz U.E., Legner C., Bulling F., Freitag L., Faßauer C., Schafföner S., Aneziris C.G. (2019). Investment casting of titanium alloys with calcium zirconate moulds and crucibles. Int. J. Adv. Manuf. Technol..

[10] Chen G., Kang J., Gao P., Qin Z., Lu X., Li C. (2018). Dissolution of BaZrO_3_ refractory in titanium melt. Int. J. Appl. Ceram. Technol..

[11] Zhang Z., Zhu K.L., Liu L.J., Lu X.G., Wu G.X., Li C.H. (2013). Preparation of BaZrO_3_ crucible and its interfacial reaction with molten titanium alloys. J. Chin. Ceram. Soc..

[12] Mamayeva A.A., Panichkin A.V., Chukmanova M.T., Imbarova A.T., Kenzhaliyev B.K., Belov V.D. (2022). Investigation of the mechanism for interaction of calcium zirconate, oxides of calcium and zirconium with titanium melts. Int. J. Cast Met. Res..

[13] Passerone A., Muolo M.L., Valenza F. (2016). Critical Issues for Producing UHTC-Brazed Joints: Wetting and Reactivity. J. Mater. Eng. Perform..

[14] Zhu J., Kamiya A., Yamada T., Shi W., Naganuma K., Mukai K. (2002). Surface tension, wettability and reactivity of molten titanium in Ti/yttria-stabilized zirconia system. Mater. Sci. Eng. A.

[15] Eustathopoulos N. (1998). Dynamics of wetting in reactive metal/ceramic systems. Acta Mater..

[16] Li J., Zhang H., Gao M., Li Q., Bian W., Tao T., Zhang H. (2018). High-Temperature Wettability and Interactions between Y-Containing Ni-Based Alloys and Various Oxide Ceramics. Materials.

[17] Barbosa J., Puga H., Ribeiro C.S., Teodoro O.M.N.D., Monteiro A.C. (2006). Characterisation of metal/mould interface on investment casting of γ-TiAl. Int. J. Cast Met. Res..

[18] Lin K.F., Lin C.C. (1999). Interfacial reactions between Ti-6Al-4V alloy and zirconia mold during casting. J. Mater. Sci..

[19] Warren J.A., Boettinger W.J., Roosen A.R. (1998). Modeling reactive wetting. Acta Mater..

[20] Meier A., Javernick D.A., Edwards G.R. (1999). Ceramic-metal interfaces and the spreading of reactive liquids. J. Manag..

[21] Ding S., Li M., Wang H., Zhu J., Shao G., Xu H., Lu H., Zhang R. (2024). Preparation of a (Ca, Sr, Ba) ZrO_3_Crucible by Slip Casting for the Vacuum Induction Melting of NiTi Alloy. Materials.

[22] Naidich Y.V., Zhuravlev V.S., Chuprina V.G. (1974). Adhesion, wetting, and formation of intermediate phases in systems composed of a titanium-containing melt and an oxide: II. Systems Ni-Ti/Al_2_O_3_ and Ni-Mo-Ti/Al_2_O_3_. Sov. Powder Metall. Met. Ceram..

[23] Wan C., Kritsalis P., Drevet B., Eustathopoulos N. (1996). Optimization of wettability and adhesion in reactive nickel-based alloys/alumina systems by a thermodynamic approach. Mater. Sci. Eng. A.

[24] Schafföner S., Fruhstorfer J., Faßauer C., Freitag L., Jahn C., Aneziris C.G. (2019). Advanced refractories for titanium metallurgy based on calcium zirconate with improved thermomechanical properties. J. Eur. Ceram. Soc..

[25] Schafföner S., Fruhstorfer J., Faßauer C., Freitag L., Jahn C., Aneziris C.G. (2017). Influence of in situ phase formation on properties of calcium zirconate refractories. J. Eur. Ceram. Soc..

[26] Freitag L., Schafföner S., Lippert N., Faßauer C., Aneziris C.G., Legner C., Klotz U.E. (2017). Silica-free investment casting molds based on calcium zirconate. Ceram. Int..

[27] Shornikov S. (2019). High-temperature Mass Spectrometry Study of the Thermodynamic Properties of CaTiO_3_ Perovskite. Russ. J. Phys. Chem..

[28] Simon A., Redfern T. (1996). High-temperature structural phase transitions in perovskite. J. Phys. Condens. Matter.

[29] Guyot F., Richet P., Courtial P., Gillet P. (1993). High-temperature heat capacity and phase transitions of CaTiO_3_ perovskite. Phys. Chem. Miner..

[30] Roushown A., Masatomo Y. (2005). Space group and crystal structure of the Perovskite CaTiO_3_ from 296 to 1720K. J. Solid State Chem..

[31] Lu M.W., Lin K., Lin C.C. (2021). Microstructural evolution and interfacial reactions between Ti and ZrO_2_/CaTiO_3_ composites. Int. J. Appl. Ceram. Technol..

[32] Bewlay B.P., McKiever J., Ellis B.M., McLasky N.V. (2014). Calcium Titanate Containing Mold Compositions and Methods for Casting Titanium and Titanium Aluminide Alloys.

[33] Li Z., Du R., Fu L., Or S.W., Gu H., Chen D., Yang S., Huang A., Lv R. (2023). Fabrication of CaAl_12_O_19_–CaTiO_3_ composites and their potential usage for TiAl alloy smelting. J. Am. Ceram. Soc..

[34] Lu M.-W., Lin K.-L., Lin C.-C. (2020). Interfacial reactions between Ti and Y_2_O_3_/Ca_4_Ti_3_O_10_ composites. Int. J. Appl. Ceram. Technol..

[35] Schafföner S., Aneziris C.G., Berek H., Rotmann B., Friedrich B. (2015). Investigating the Corrosion Resistance of Calcium Zirconate in Contact with Titanium Alloy Melts. J. Eur. Ceram. Soc..

[36] Saha R.L., Nandy T.K., Misra R.D.K., Jacob K.T. (1990). On the Evaluation of Stability of Rare Earth Oxides as Face Coats for Investment Casting of Titanium. Metall. Trans. B.

[37] Bulling F., Klotz U.E., Heiss A., Freitag L., Faßauer C., Aneziris C.G. (2024). Towards High-Quality Investment Casting of Ti-6Al-4V with Novel Calcium Zirconate Crucibles and Optimized Process Control. Metals.

[38] Obinata I., Takeuchi Y., Saikawa S. (1960). The System Titanium-Calcium. Trans. Am. Soc. Met..

[39] (2002). Ca-Ti Phase Diagrams as Published.

[40] Gralik G., Thomsen A.E., Moraes C.A., Raupp-Pereira F., Hotza D. (2014). Processing and characterization of CaTiO_3_ perovskite ceramics. Process. Appl. Ceram..

[41] Jacob K.T., Srikanth S., Waseda Y. (1988). Activities, Concentration Fluctuations and Complexing in Liquid Ca–Al Alloys. Trans. Jpn. Inst. Met..

[42] Shornikov S., Lambotte G., Lee J., Allanore A., Wagstaff S. (2019). High-Temperature Study of Perovskite Evaporation. Materials Processing Fundamentals.

[43] Okamoto H. (2011). O-Ti (oxygen-titanium). J. Phase Equilibria Diffus..

